# Supplementary material for: AKAP150-anchored PKA regulates synaptic transmission and plasticity, neuronal excitability and CRF neuromodulation in the mouse lateral habenula
Source: Commun Biol. 2024 Mar 20;7:345. doi: 10.1038/s42003-024-06041-8 (PMC10954712; doi:10.1038/s42003-024-06041-8)
Supplement: Supplementary file 2 — Supplementary Information [file 42003_2024_6041_MOESM2_ESM.pdf]

Supplemental Figure 1.

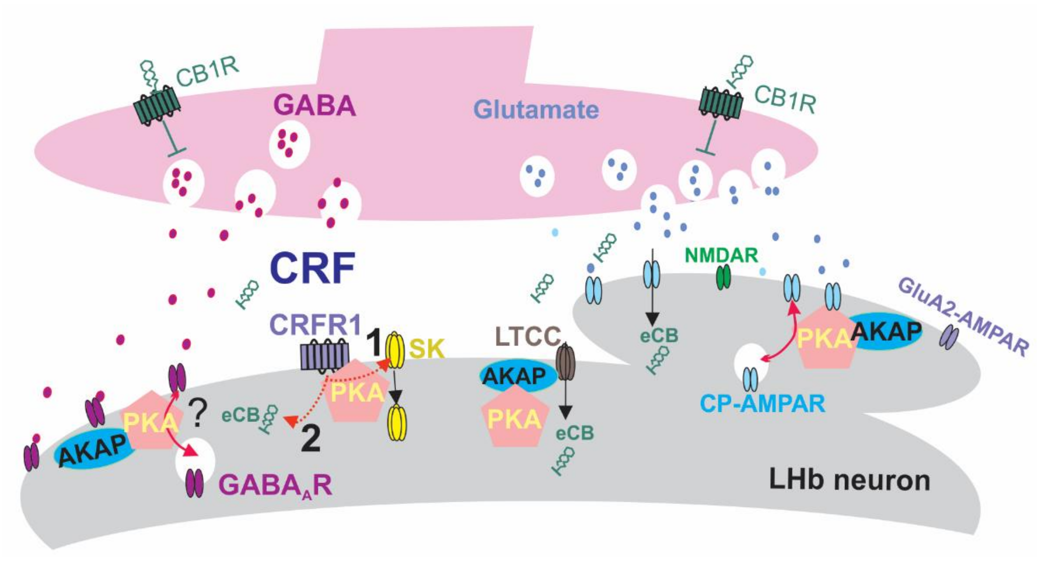

Schematic proposed model illustrating regulatory roles of AKAP150-anchored PKA signaling in synaptic function, LTD and intrinsic excitability of LHb neurons. This model predicts that AKAP150-PKA association is necessary for maintenance of AMPARs at glutamatergic synapses and expression of LTD by LFS. We assume that the sources of increased depolarization and calcium influx for eCB production during LTD induction may arise from CP-AMPARs and/or LTCC both regulated by AKAP150-PKA complex. Therefore, disruption of AKAP150 anchoring of PKA to CP-AMPARs in LHb neurons in ∆PKA mice results in lower number of CP-AMPARs at glutamatergic synapses. The reduced influx of calcium from fewer available CP-AMPARs at the synapse as well as hypofunctional LTCC by the genetic disruption of PKA-AKAP150 association can impair eCB production and thus the expression of eCB-LTD. Our model also predicts that defective AKAP150-PKA association may reduce trafficking and/or function of potassium channels mediating mAHPs (e.g., M currents, not shown). The model also shows the known effects of CRF-CRF receptor 1-PKA signaling in the LHb

which results in eCB production as well as promote LHb hyperexcitability through modulation of trafficking or conductance of SK potassium channels. Thus, we assume that under pathological conditions AKAP150-PKA dysregulation of CP-AMPARs, LTCC, M currents and eCB signaling could promote LHb hyperexcitability and blunt CRF neuromodulatory actions. While our model may indicate the existence of inhibitory effects of AKAP150-PKA on $GABA_AR$ feedforward trafficking, it is still unclear which AKAP150 partners may contribute to regulation of $GABA_ARs$ in LHb neurons. The ? indicates such unresolved questions.
