# Supplementary material for: AKAP150-anchored PKA regulates synaptic transmission and plasticity, neuronal excitability and CRF neuromodulation in the mouse lateral habenula
Source: Commun Biol. 2024 Mar 20;7:345. doi: 10.1038/s42003-024-06041-8 (PMC10954712; doi:10.1038/s42003-024-06041-8)
Supplement: Supplementary file 3 — Description of Supplementary Materials [file 42003_2024_6041_MOESM3_ESM.docx]

**Description of Additional Supplementary Files**

**File name:** Supplementary Data 1

**Description:** The source data behind the graphs.
